# First-in-human study of TK-positive oncolytic vaccinia virus delivered by adipose stromal vascular fraction cells

**DOI:** 10.1186/s12967-019-2011-3

**Published:** 2019-08-19

**Authors:** Boris R. Minev, Elliot Lander, John F. Feller, Mark Berman, Bernadette M. Greenwood, Ivelina Minev, Antonio F. Santidrian, Duong Nguyen, Dobrin Draganov, Mehmet O. Killinc, Anna Vyalkova, Santosh Kesari, Edward McClay, Gabriel Carabulea, Francesco M. Marincola, Lisa H. Butterfield, Aladar A. Szalay

**Affiliations:** 1 Calidi Biotherapeutics, 10210 Campus Point Drive, Suite 150, San Diego, CA 92121 USA; 2 Department of Radiation Medicine and Applied Sciences, Moores UCSD Cancer Center, San Diego, USA; 3 Cell Surgical Network, Beverly Hills, USA; 4 Desert Medical Imaging, Palm Desert, USA; 5 Institute of Biochemistry, University of Wurzburg, Würzburg, Germany; 6 Department of Translational Neurosciences and Neurotherapeutics, John Wayne Cancer Institute, Santa Monica, USA; 7 California Cancer Associates for Research and Excellence, San Diego, USA; 8 Ocean View Hematology and Oncology Medical Group, San Clemente, USA; 9 Refuge Biotechnologies, Menlo Park, CA USA; 10 University of Pittsburgh, UPMC Hillman Cancer Center, Pittsburgh, USA

**Keywords:** Clinical trial, Oncolytic vaccinia virus, Stromal vascular fraction, Immunotherapy of cancer

## Abstract

**Background:**

ACAM2000, a thymidine kinase (TK)-positive strain of vaccinia virus, is the current smallpox vaccine in the US. Preclinical testing demonstrated potent oncolytic activity of ACAM2000 against several tumor types. This Phase I clinical trial of ACAM2000 delivered by autologous adipose stromal vascular fraction (SVF) cells was conducted to determine the safety and feasibility of such a treatment in patients with advanced solid tumors or acute myeloid leukemia (AML).

**Methods:**

Twenty-four patients with solid tumors and two patients with AML participated in this open-label, non-randomized dose-escalation trial. All patients were treated with SVF derived from autologous fat and incubated for 15 min to 1 h with ACAM2000 before application. Six patients received systemic intravenous application only, one patient received intra-tumoral application only, 15 patients received combination intravenous with intra-tumoral deployment, 3 patients received intravenous and intra-peritoneal injection and 1 patient received intravenous, intra-tumoral and intra-peritoneal injections. Safety at each dose level of ACAM2000 (1.4 × 10^6^ plaque-forming units (PFU) to 1.8 × 10^7^ PFU) was evaluated. Blood samples for PK assessments, flow cytometry and cytokine analysis were collected at baseline and 1 min, 1 h, 1 day, 1 week, 1 month, 3 months and 6 months following treatment.

**Results:**

No serious toxicities (> grade 2) were reported. Seven patients reported an adverse event (AE) in this study: self-limiting skin rashes, lasting 7 to 18 days—an expected adverse reaction to ACAM2000. No AEs leading to study discontinuation were reported. Viral DNA was detected in all patients’ blood samples immediately following treatment. Interestingly, in 8 patients viral DNA disappeared 1 day and re-appeared 1 week post treatment, suggesting active viral replication at tumor sites, and correlating with longer survival of these patients. No major increase in cytokine levels or correlation between cytokine levels and skin rashes was noted. We were able to assess some initial efficacy signals, especially when the ACAM2000/SVF treatment was combined with checkpoint inhibition.

**Conclusions:**

Treatment with ACAM2000/SVF in patients with advanced solid tumors or AML is safe and well tolerated, and several patients had signals of an anticancer effect. These promising initial clinical results merit further investigation of therapeutic utility.

*Trial registration* Retrospectively registered (ISRCTN#10201650) on October 22, 2018.

## Background

Due to the vast knowledge expansion in tumor immunology in recent years, immunotherapy is quickly becoming a major cancer treatment modality [1]. At the same time, several major obstacles to the successful cancer immunotherapy are emerging. Immune suppressive microenvironment at tumor sites is a significant hindrance, leading to limited responsiveness to modern immunotherapeutic agents in many tumor types [2]. These “cold” tumors are lacking infiltration of Th1-polarized immune effector cells and this characteristic is generally associated with poor clinical prognosis [3, 4, 5] and diminished likelihood of responding to immunotherapy such as checkpoint inhibitor therapy [6].

A promising strategy to activate the immune system at the tumor sites is the use of oncolytic viruses. Many recent studies are confirming the ability of oncolytic viruses to enhance immune cell infiltration, thus converting “cold” tumors into “hot” tumors, potentially leading to a better responsiveness to the current combination immunotherapy approaches [7–9].

Currently, a number of oncolytic viruses including vaccinia virus, herpes simplex virus-1, adenovirus, ECHO-7, seneca valley virus, reovirus and other viruses are at various stages of clinical development [10]. The ideal oncolytic virus would be very safe to treat even severely immuno-suppressed cancer patients, would have potent anti-tumor properties against multiple tumor types, and would be able to attack and kill all tumor cells at distant metastatic sites. It would be easily manufactured and stored for widespread use. Further, the need for genetic manipulation of the virus would be minimal. None of the viruses currently under investigation has the ability to fulfill this ideal viral phenotype. However, the oncolytic vaccinia virus may satisfy many of the above criteria.

Vaccinia virus (VV) is a large and complex particle containing a single linear double-stranded DNA genome of approximately 190 kb, encoding approximately 150–200 proteins [11]. This virus has many characteristics desirable in an oncolytic virus for clinical applications: (1) short, well-characterized life cycle, spreading very rapidly from cell-to-cell, (2) highly cytolytic for a broad range of tumor cell types, (3) a large insertion capacity (> 25 kb) for the expression of exogenous genes if required, (4) high genetic stability, (5) amenable to large scale production of high levels of infectious virus; (6) does not cause any known diseases in humans, (7) remains in the cytoplasm and does not enter the host cell nucleus during the entire life cycle, and thus does not integrate into the host genome, (8) used extensively as smallpox vaccine in millions of people with well documented safety profile [12], (9) drugs (e.g. vaccinia immunoglobulin, cidofovir, etc.) are available to effectively treat any potential vaccinia infections, and (10) has previously been safely administered to patients with advanced cancer [13] [14].

Majority of known VV strains were used as vaccines in the World Health Organization Smallpox Eradication Program (1966–1980). In the US, the main VV strain used was Dryvax—used as smallpox vaccine until 2008 when it was substituted by ACAM2000 (Acambis, Inc.™), a single plaque-purified vaccinia virus derivative of Dryvax [15, 12], selected based on its reduced virulence [16, 17].

ACAM2000 genome carries key genomic alterations that explain its reduced virulence [18]. Two main disrupted factors are immunomodulatory: (i) a tumor necrosis factor receptor, and (ii) the interferon α/β binding protein [18]. Moreover, ACAM2000 genome presents other genomic alterations that might contribute to its naturally occurring reduced virulence, namely a truncation of ankyrin-repeat ortholog of a VARV (Variola)-and CPXV (cowpox)-like protein and a short form of the thymidylate kinase gene [18].

To reduce the risk of therapeutic use of vaccinia virus, highly virulent vaccinia virus strains like Western Reserve have been genetically-engineered to attenuate the virus and to improve safety [19–21]. Elimination of TK gene from the virulent Western Reserve strain decreased significantly the lethality of mice injected intra-cranially with the virus [21]. Therefore, the TK gene from vaccinia virus genome became the primary target to attenuate highly virulent viruses or viruses with unknown virulence. In contrast, the ACAM2000 derivation exemplifies an alternative strategy to generate a safer vaccinia virus: selection of a naturally occurring clone with a significantly reduced virulence and improved safety profile. Therefore, the TK-positive ACAM2000 possesses both strong anti-tumor activities associated with unattenuated viruses and a significant safety profile due to the natural clonal selection.

Although the safety profile of ACAM2000 is well established, a possible concern for the use of this virus in cancer patients is increased toxicity due to virus amplification within the tumor and cancer-related immunosuppression as commonly observed particularly in advanced stage patients [22].

In preclinical studies we confirmed that ACAM2000 is a very potent oncolytic virus, able to infect and kill multiple human cancer cells lines in vitro (Table 1). However, we and others confirmed that the complement system can neutralize most of the viral particles after intravenous deployment [23]. Therefore, we suggested that the viral particles taken up by autologous SVF cells would be protected from the patients’ immune system, thus allowing effective delivery to the tumor sites. In addition, SVF contains stem cells exhibiting a natural tropism towards tumor sites, which could be exploited to transport the viral payloads directly to the tumor sites [24]. Therefore, we designed a clinical trial utilizing autologous SVF cells incubated with vaccinia virus (ACAM2000/SVF) in patients with advanced solid tumors or AML. The current study is a first-in-human trial to determine the safety and feasibility of this approach in patients with advanced solid cancers or AML.

**Table 1:** Oncolysis of a panel of human tumor cell lines after infection with ACAM2000

Hours post Infection	Human tumor cell line (percent viability)			
	PC3	DU145	MDA-MB-231	A549
24	67	100	85	80
48	64	80	63	45
72	33	66	36	41
96	13	51	21	30

## Methods

### Therapeutic efficacy of ACAM2000 in vitro

We analyzed the anticancer therapeutic efficacy of ACAM2000 in vitro against human prostate cancer cell lines PC3 and DU145, triple negative breast cancer cells MDA-MB-231 and lung adenocarcinoma line A549. Tumor cells were seeded on 96-well plates 24 h prior to infection. The following day, cells were almost confluent and were infected with 50 μl of DMEM media supplemented with 2% fetal bovine serum (FBS) containing appropriate amount of ACAM2000 (MOI of 1). Control cells were left untreated. After 1 h of incubation the media were replaced with DMEM supplemented with 10% FBS. Tumor cell death was then analyzed 24, 48, 72 and 96 h post treatment by MTT assay as follows. At 24, 48, 72, or 96 h after infection of cells, medium was replaced by 100 uL MTT solution at a concentration of 2.5 mg/ml MTT dissolved in DMEM and incubated for 2 h at 37 °C in a 5% CO_2_ atmosphere. After removal of the MTT solution, the color reaction was stopped by adding 150 uL 1 N HCl diluted in isopropanol. The optical density was then measured at a wavelength of 570 nm with a reference wavelength of 650. Uninfected cells were used as reference and were considered as 100% viable.

### Clinical trial

This open-label, non-randomized dose-escalation trial was approved by the International Cell Surgical Society Institutional Review Board. Written informed consent was obtained from all patients prior to treatment, and the study was conducted in compliance with Good Clinical Practice guidelines.

### Eligibility

Patients (≥ 18 years) were required to have a histologically proven, primary or recurrent, advanced (staging defined by the *American Joint Committee on Cancer* (AJCC; 7th Edition) stage III or IV, and/or aggressive (defined as published disease-specific survival rates less than 20% at 5 years following best currently available therapies) solid organ cancers. Two IRB exemptions were made to include two patients with AML. All patients had to be able to understand and be willing to sign a written informed consent. They had to have no continuing acute toxic effects of any prior therapy, including but not limited to biological therapy, radiotherapy, chemotherapy, or surgical procedures, i.e., all such effects must have resolved to *Common Terminology Criteria for Adverse Events* (CTCAE, Version 4.0) Grade ≤ 1. Any other surgery (except biopsies) must have occurred at least 28 days prior to study enrollment. ECOG performance Status of 0 to 2 was acceptable. A life expectancy of at least 3 months was required. Also, adequate organ and marrow function was required, as follows: Absolute neutrophil count (ANC) ≥ 1.5 × 10^9^; Platelets ≥ 100 × 10^9^ (without platelet transfusion); Hemoglobin ≥ 9.0 g/dL (with or without red blood cell (RBC) transfusion); Serum creatinine ≤ 1.5 × upper limit of normal (ULN); Bilirubin ≤ 1.5 × ULN; ALT and AST at ≤ 2.5 × ULN (in case of liver metastasis AST/ALT at ≤ 5.0 × ULN); LDH ≤ 1.5 × ULN. Women of child-bearing potential and men with partners of child-bearing potential had to agree to use adequate contraception (hormonal or barrier method of birth control; abstinence) prior to study entry, for the duration of study participation, and for 90 days following completion of therapy. Women of child-bearing potential had to have negative pregnancy test prior to initiating study drug dosing. All patients had to be willing and able to comply with scheduled visits, the treatment plan, imaging and laboratory tests.

Excluded from the study were all patients with a current or anticipated use of other investigational agents or marketed anticancer agents. Also excluded were patients who have received chemotherapy or radiotherapy within 4 weeks prior to entering the study or has not recovered from adverse events due to agents administered more than 4 weeks earlier. Other exclusion criteria included immune system disorders (including acquired immunodeficiency syndrome (AIDS), HIV infection or hepatitis B or C); Patients who were receiving additional immunosuppressive therapy or any steroids (except concurrent corticosteroid usage if no more than 20 mg per day, prednisolone equivalent is applied); Patients who have received prior gene therapy or therapy with cytolytic virus of any type; Patients with clinically significant cardiac disease (New York Heart Association Class III or IV) including pre-existing arrhythmia, uncontrolled angina pectoris, and myocardial infarction 1 year prior to study entry, or Grade 2 or higher compromised left ventricular ejection fraction; Patients with dementia or altered mental status that would prohibit informed consent; patients with severe or uncontrolled medical disorder that would, in the investigator’s opinion, impair ability to receive study treatment (i.e., uncontrolled diabetes, chronic renal disease, chronic pulmonary disease or active, fever, systemic and/or uncontrolled infections, psychiatric illness/social situations that would limit compliance with study requirements); patients receiving concurrent antiviral agent active against vaccinia virus (e.g., cidofovir, vaccinia immunoglobulin, imatinib, ST-246) during the course of study; patients with known allergy to ovalbumin or other egg products; patients with clinically significant dermatological disorders (e.g., eczema, psoriasis, or any unhealed skin wounds or ulcers) as assessed by the Principal Investigator during screening and during the study; patients with a history of allergy to iodinated contrast media; patients with an active dental infection or recent dental work within 2 weeks of deployment; patients with known brain metastases were excluded from this clinical trial because of their poor prognosis and because they often develop progressive neurologic dysfunction that would confound the evaluation of neurologic and other adverse events.

### Treatment

Patients were admitted to the outpatient clinic and a mini-liposuction procedure was performed to isolate up to 100 milliliters of adipose tissue. All patients received local anesthesia consisting of lidocaine 0.5% with epinephrine 1:400,000 with HCO_3_ 8.4% titrated to pH of 7.4. Then sterile prep was performed, followed by liposuction procedure utilizing the Time-Machine™ device, fat processing unit (syringe) and 2.5–3 mm cannula. Bacitracin ointment and a small bandage were secured over the wound along with a compressive bandage. The SVF cells were prepared in a closed system according to an established protocol [25]. Specific amounts of isolated adipose tissue, numbers of isolated SVF cells and their viability levels are listed in Table 2.

**Table 2:** Isolation of adipose tissue and characterization of SVF cells

Patient ID	Volume of adipose tissue (ml)	Total number of SVF cells	Viability of SVF cells (%)
1	40	34 × 10^6^	76
3	50	34 × 10^6^	87
4	50	196 × 10^6^	62
5	50	67 × 10^6^	95
8	30	29 × 10^6^	94
10	50	214 × 10^6^	86
14	50	19 × 10^6^	78
15	100	42 × 10^6^	86
18	40	30 × 10^6^	96
21	50	216 × 10^6^	92
22	51	46 × 10^6^	91
23	50	80 × 10^6^	93
24	51	147 × 10^6^	88
25	50	20 × 10^6^	65
26	50	59 × 10^6^	70
27	29	14 × 10^6^	82
28	90	9.4 × 10^6^	46
29	50	49 × 10^6^	84
30	90	32 × 10^6^	78
31	10	54 × 10^6^	75
32	50	20 × 10^6^	75
33	50	32 × 10^6^	77
34	50	66 × 10^6^	60
35	50	114 × 10^6^	86
36	50	94 × 10^6^	79
47	50	46 × 10^6^	47

Patients’ adipose tissue was obtained aseptically in the operating room and processed in sterile conditions to isolate the SVF cells

The ACAM2000 vaccine was prepared according to manufacturer’s protocol. After reconstitution of the lyophilized preparation, each vial contained approximately 2.5–12.5 × 10^7^ plaque-forming units (pfu) of live vaccinia virus. Specific amount of ACAM2000 vaccine was added to a labeled 20 cc syringe containing the SVF cells to achieve multiplicity of infection (MOI) = 1. The syringe was then placed on a rotator inside a 37° incubator, and was incubated for 15 min to 1 h with constant rotation at 20 rpm.

Twenty-four patients with solid tumors and two patients with AML received a single treatment with ACAM2000/SVF. Six patients received systemic intravenous application only, one patient received intratumoral application only, 15 patients received combination intravenous with intra-tumoral deployment, 3 patients received intravenous and intra-peritoneal injection and 1 patient received intravenous, intra-tumoral and intra-peritoneal injections. To confirm the safety and tolerability of ACAM2000/SVF treatment, a dose escalation was incorporated into the study design. The dose range for ACAM2000 in this trial was between 1.4 × 10^6^ and 1.8 × 10^7^ pfu.

### Deployment methods

Specific doses of injected SVF cells loaded with ACAM2000 at each route of administration are listed in Table 3.

**Table 3:** Routes of administration and dose of injected SVF cells loaded with ACAM2000

Patient ID	Route of administration and dose (SVF cell numbers)			
	I.V.	I.T.	I.P.	Total
1	2.5 × 10^6^			2.5 × 10^6^
3	1.4 × 10^6^	1.6 × 10^6^		3 × 10^6^
4	3 × 10^6^			3 × 10^6^
5	3 × 10^6^	3 × 10^6^		6 × 10^6^
8	4.6 × 10^6^	2.4 × 10^6^		7 × 10^6^
10	1.2 × 10^7^	6 × 10^6^		1.8 × 10^7^
14	1.5 × 10^6^		1.5 × 10^6^	3 × 10^6^
15	3.1 × 10^6^	4.8 × 10^5^		3.6 × 10^6^
18	3 × 10^6^			3 × 10^6^
21	2 × 10^6^	1 × 10^6^		3 × 10^6^
22	1 × 10^6^	2 × 10^6^		3 × 10^6^
23	3 × 10^6^		3.6 × 10^6^	6.6 × 10^6^
24	6.6 × 10^6^		6.6 × 10^6^	1.3 × 10^7^
25	1 × 10^6^	2 × 10^6^		3 × 10^6^
26	2.1 × 10^6^	9 × 10^5^		3 × 10^6^
27	2.1 × 10^6^	9 × 10^5^		3 × 10^6^
28	2.1 × 10^6^	9 × 10^5^		3 × 10^6^
29	3 × 10^6^			3 × 10^6^
30	8.4 × 10^5^	5.6 × 10^5^		1.4 × 10^6^
31	7.5 × 10^5^	6.5 × 10^5^		1.4 × 10^6^
32	7 × 10^5^	7 × 10^5^		1.4 × 10^6^
33	6.5 × 10^5^	5.4 × 10^5^	2.2 × 10^5^	1.4 × 10^6^
34	3 × 10^6^			3 × 10^6^
35	7 × 10^5^	7 × 10^5^		1.4 × 10^6^
36	1.4 × 10^6^			1.4 × 10^6^
47		5 × 10^6^		5 × 10^6^

Patients’ SVF cells were isolated and incubated with ACAM2000 at a ratio of 1:1 (MOI 1) before the I.V., I.T. and I.P. injections

- Intravenous: the non-expanded, autologous stromal vascular fraction (SVF) extracted from up to 100 ml of lipoaspirate and purified by collagenase digestion and a series of washing steps and containing up to 100 million cells incubated with vaccinia virus was delivered by intravenous injection in a volume of 20 mL.
- Intra-tumoral: the SVF incubated with vaccinia virus was delivered by intra-tumoral injection at the investigator’s clinical site or at Desert Medical Imaging with CT guidance or MRI guidance. The injection volume and number of injected cells varied depending on tumor type and tumor size and location.
- Intra-peritoneal: the SVF incubated with vaccinia virus was delivered by intra-peritoneal injection at the investigator’s clinical site or at Desert Medical Imaging with ultrasound guidance. The injection volume and number of injected cells varied depending on tumor type.

### Safety assessments

A complete medical history was taken and a physical examination performed at screening.

Adverse events were monitored throughout the study until resolution. In addition, all patients responded to a weekly questionnaire answering specific questions on their current condition.

### Pharmacokinetics of ACAM2000

We analyzed the pharmacokinetics of ACAM2000 by quantifying the amount of viral DNA present in the peripheral blood of patients treated with ACAM2000/SVF by quantitative PCR (qPCR). Specifically, 0.5–5 ml peripheral blood from the treated patients were collected in Vacutainer K2EDTA blood collection tubes (Becton, Dickinson, NJ) before treatment, 1 min, 60 min, 1 day, 1 week, 1 month, 3 month and 6 months after treatment. DNA was extracted using the Quick-gDNA™ Blood MidiPrep (Zymo Research, CA). The copy number amount of the ACAM2000 gene A56R was quantified by qPCR using PowerUp™SYBR^®^ Green Master Mix (Thermofisher, CA) and the following primers: A56R-F CAT (CAT CTG GAATTG TCA CTA CTA AA), A56R-R (ACG GCC GAC AAT ATA ATT AAT GC) described previously by Dr. Damon group at Poxvirus and Rabies Branch, Division of Viral and Rickettsial Diseases National Center for Zoonotic, Vector-Borne and Enteric Diseases, Centers for Disease Control and Prevention (CCID/CDC) [26]. A pUC57 plasmid containing a single copy of A56R open reading frame from ACAM2000 was created (Vectorbuilder—Cyagen Biosciences Inc, CA, USA) for use as a positive control and to generate a standard curve for the qPCR assays. Data were recorded and analyzed using an ABI-PRISM 7700 Sequence Detection System (Applied Biosystems) and Sequence Detector Software (SDS v2.2).

### Flow cytometry and cytokine analysis

Blood samples for PBMC Flow Cytometry and Cytokine analysis were collected in Vacutainer Heparin blood collection tubes at Baseline and 1 day, 1 week, 1 month, 3 months and 6 months following the treatment. PBMC were isolated by Ficoll-Paque method. Plasma samples for cytokines analysis were isolated by centrifugation of blood sample. All PBMC and Plasma samples were sent for analysis to the Immunologic Monitoring Laboratory (IML) at the University of Pittsburgh Cancer Institute. The IML has an independently monitored and extensive quality control (QC) and quality assurance (QA) program to ensure the validity of test results and safety/quality of therapeutic products, and participates in external proficiency panels. The IML also serves as the Central Immunology Laboratory for the Eastern Cooperative Oncology Group (ECOG). All activities were carried out in accordance with Good Laboratory Practices as outlined in Title 21 of the U.S. Code of Federal Regulations Part 58, using Standard Operating Procedures (SOP) and including appropriate quality control.

### Efficacy evaluation

Although this Phase I clinical study was designed to establish the safety and tolerability of ACAM2000/SVF, we were able to assess some initial efficacy signals following treatment with the ACAM2000/SVF combination. Brief case reports for two of the responding patients are presented in the results section. Most patients were not evaluable by response evaluation criteria for solid tumors (RECIST) criteria, because all of their tumors were not measured over time.

### Statistical methods

Descriptive statistics for the safety and efficacy assessments were calculated and displayed by group. Kaplan-Meyer curves displaying the percentage of patients who survived after a given number of months were calculated for various groups in order to detect whether the treatment with ACAM2000/SVF had an effect on survival. Patients who dropped out of the study before it was completed were censored from the calculation of the survival curves. The set of curves were for patients with: (a) present skin rashes; (b) persistent viral DNA in the blood, and (c) for patients who had both persistent viral DNA in the blood and skin rashes (Fig. 3). For each group of patients, both a Gehan-Breslow-Wlcoxon and Mantel-Cox t-tests were used to determine any degree of statistical significance, and the median survival rates were calculated for all groups.

## Results

### ACAM2000 is effective against multiple human tumor cell lines in vitro

ACAM2000 virus strain killed all tested human cancer cell lines efficiently, including prostate cancer cell lines PC-3 and DU154, triple negative breast cancer cells MDA-MB-231 and lung adenocarcinoma line A549 (Table 1).

### Patient demographics

As shown in Table 4, twenty-six patients were enrolled in this study—15 male and 11 female patients. Twenty-four patients with 15 different solid tumor types and 2 patients with AML were enrolled.

**Table 4:** Demographics

Age (years)		
Median	60.4	
Range	19–92	
Gender	N	%
Male	15	58
Female	11	42
Cancer type	N	%
Lung cancer	1	4
Breast cancer	2	8
Prostate cancer	3	12
AML	2	8
Pancreatic cancer	2	8
Colorectal cancer	2	8
Head and Neck cancer	4	15
Adrenal cancer	1	4
Liver cancer	1	4
Astrocytoma	1	4
Bronchial carcinoid	1	4
Ovarian cancer	1	4
Uterine cancer	1	4
Sarcomatoid	1	4
Squamous cell carcinoma neck	1	4
Esophageal cancer	1	4
Thyroid cancer	1	4
Route of administration	N	%
Intravenous (IV)	6	23
Intravenous + Intratumoral	15	58
Intratumoral (IT)	1	4
Intraperitoneal (IP) +IV	3	12
IV + IT + IP	1	4

### Toxicity

As shown in Table 5, self-limiting skin rashes and small lipo-puncture bleeding were the only treatment-related adverse events in this study. These self-limiting skin rashes are an expected adverse reaction to the ACAM2000 administration. No other treatment-related AEs were reported. No infusion-related AEs were reported. No AEs leading to study discontinuation were reported. There was no apparent dose-dependent effect in the type or severity of AEs that occurred following ACAM2000/SVF treatment. Three patients had presence of viral DNA in peripheral blood 1 month after treatment without experiencing any AE.

**Table 5:** Adverse events

Adverse events	Number	%	Related	Resolved
Skin rashes	8	40	Yes	Yes
Lipo- puncture bleeding	1	5	Yes	Yes
Fever (100.5 F)	2	10	No (10 days and 16 days a/t)	Yes
Pain	4	20	No (7 days, 10 days and 1 month a/t)	Yes
Hemoptysis	1	5	No (2 months a/t)	Yes
Pleural effusion	1	5	No (3 months a/t)	Yes
Headache and weakness of one side	1	5	No (3 weeks a/t)	Yes
Blood Transfusion	1	5	No (10 days a/t)	Yes
Pneumonia	1	5	No (16 days a/t)	Yes

a/t: After treatment; pt: patient

Another important finding of this study was that 18 patients experienced virus-related and inflammation-related symptoms at the tumor sites approximately 2 weeks after treatment. Specifically, patients described this effect as “burning sensation”, fullness, heaviness and warmness, pointing at their respective tumor/metastatic sites—a condition lasting 2–4 weeks. These symptoms were self-limiting and did not require any treatment.

### Flow cytometry and cytokine analysis

Flow cytometry results: Flow cytometry data on patient-derived PBMCs indicated that treatment resulted in delayed changes that occurred gradually and over extended periods of time post treatment and without any dramatic immediate effects that can be linked to potential serious side effects or toxicities (Table 6).

**Table 6:** Representative analysis of main cell populations in patients

Patient ID	T_CD4_			T_CD8_			NK		
	B	1 w	1 m	B	1 w	1 m	B	1 w	1 m
3	24.8	20.0	27.4	20.1	20.1	27.2	8.9	–	10.4
5	8.0	3.4	5.6	3.9	2.6	2.6	19.9	13.5	23.4
8	–	12.9	14.1	–	8.5	10.2	–	3.1	6.6
10	13.4	13.6	9.6	8.5	18.7	17.5	7.3	–	–
14	30.8	35.7	36.9	32.3	31.0	28.6	5.0	3.7	3.1
15	12.6	9.2	79.9	7.8	8.0	7.3	16.3	12.6	13.9
18	12.9	11.6	13.6	4.1	8.8	5.7	11.1	3.6	10.3
21	13.8	17.9	14.6	3.4	4.1	4.0	17.6	15.9	21.7
24	49.0	32.3	51.0	6.0	3.7	6.1	6.9	3.3	5.7
26	15.0	16.6	14.1	15.9	16.3	13.7	20.9	21.0	16.1
27	22.4	19.1	8.9	9.3	11.5	3.8	4.8	10.3	6.4
28	48.2	36.4	41.9	5.2	6.5	5.6	–	8.6	6.5
29	0.4	0.6	0.6	1.2	2.1	1.7	1.6	2.2	1.7
30	22.6	22.9	23.7	8.6	7.1	14.3	6.3	6.0	6.4

Patient ID	MDSC			Tem			Tcm		
	B	1 w	1 m	B	1 w	1 m	B	1 w	1 m
3	0.6	0.6	–	1.8	2.1	2.9	1.5	1.6	2.3
5	1.8	2.3	2.3	0.5	0.3	0.5	0.5	0.3	0.3
8	–	2.7	2.1	–	3.6	3.7	–	0.3	0.3
10	1.6	–	2.0	0.5	–	2.3	0.2	–	0.4
14	0.5	0.8	0.3	3.8	3.5	3.1	19.8	17.1	14.8
15	1.1	2.0	1.2	2.1	2.7	2.3	0.5	0.6	0.8
18	2.0	1.9	3.5	0.9	3.2	1.8	0.0	0.1	0.0
21	1.7	2.4	1.7	1.2	1.3	1.4	0.0	0.1	0.1
24	0.3	0.9	0.9	0.5	0.4	0.4	1.2	0.7	1.1
26	0.5	0.7	1.1	2.3	2.6	2.2	0.1	0.2	0.2
27	1.2	0.6	0.4	3.4	3.3	1.2	0.1	0.1	0.1
28	0.5	0.7	0.4	3.3	4.2	4.3	0.2	0.2	0.2
29	9.0	5.1	6.0	0.1	0.2	0.2	0.1	0.2	0.1
30	0.3	0.2	0.2	3.0	2.0	4.3	0.5	0.5	0.5

Patients’ blood samples were analyzed by flow cytometry at different time-points after treatment: B: baseline; 1 w: 1 week post treatment; 1 m: 1 month post treatment. Data represent percent of total PBMC. Cell populations: T_CD4_ (CD3+ CD4 +), T_CD8_ (CD3+ CD8 +), NK (CD3−CD56 +), MDSC (Lin-DR-CD3 + CD116+), Tem (CD3+ CD8+ CD45RA-CD197−), Tcm (CD3+ CD8+ CD45RA−CD197+)

Cytokine analysis results: Analysis of the concentrations of 30 cytokines in the plasma of treated patients did not reveal any significant and consistent increase in cytokine levels post treatment. Particular attention was given to IL-6 levels, as this cytokine has already been linked to life threatening toxicities during cytokine release syndrome (CRS). Higher baseline IL-6 levels were observed in some patients but these remained under 1000 pg/ml far below the 10,000 pg/ml seen in CRS patients, and were not further increased following therapy. Il-6 levels did increase over time in some patients with lower baseline levels of IL-6 but these changes occurred gradually and similar to the observed gradual increase in VEGF or HGF levels likely reflecting corresponding increases in tumor burden/progression. Interestingly strong trends towards increased plasma levels of cytokines and chemokines associated with protective anti-tumor immunity was also evident including: IL-1b, IL-12, MIP1a, MIP1b, MCP-1, IL-15, IFNγ, IFNα, IL-1R, IP-10, MIG, IL-8.

**Fig. 1:**
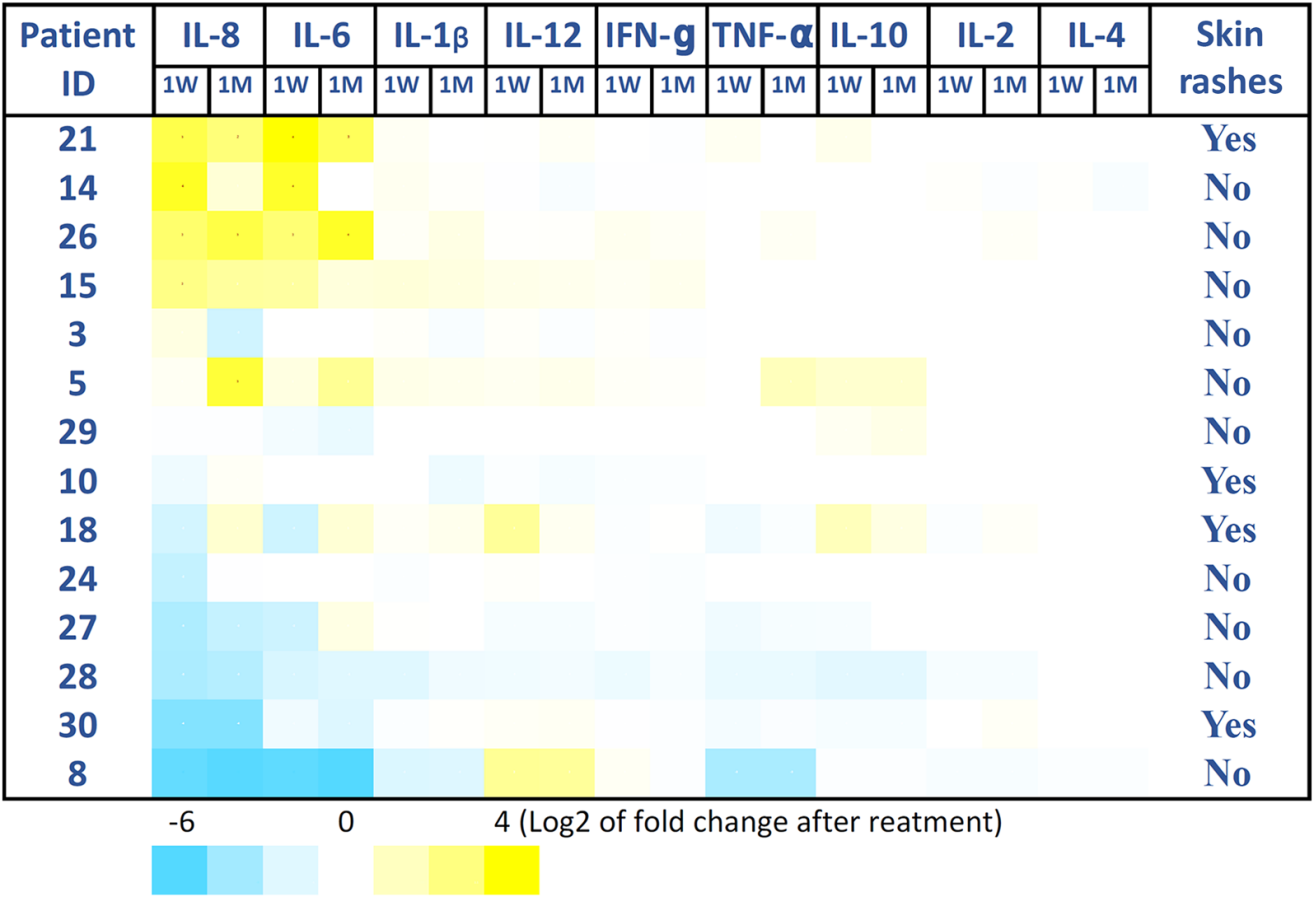
Representative analysis of 9 cytokines and the relationship with the appearance of skin rashes in patients. Patients’ plasma samples were analyzed using The Cytokine Human Magnetic 30-Plex Panel for the Luminex™ platform (Thermo Fisher) at different time-points after treatment: 1 W: 1 week post treatment; 1 M: 1 month post treatment. Data is presented as Log2 of fold change after treatment

All these responses, however, were delayed and took approximately 3 months to develop, and therefore cannot be associated with any immediate cytokine release syndrome features or toxic side effects. Representative analysis of 9 cytokines and the relationship with the appearance of skin rashes in patients is presented on Fig. 1.

### Pharmacokinetics of ACAM2000

**Fig. 2:**
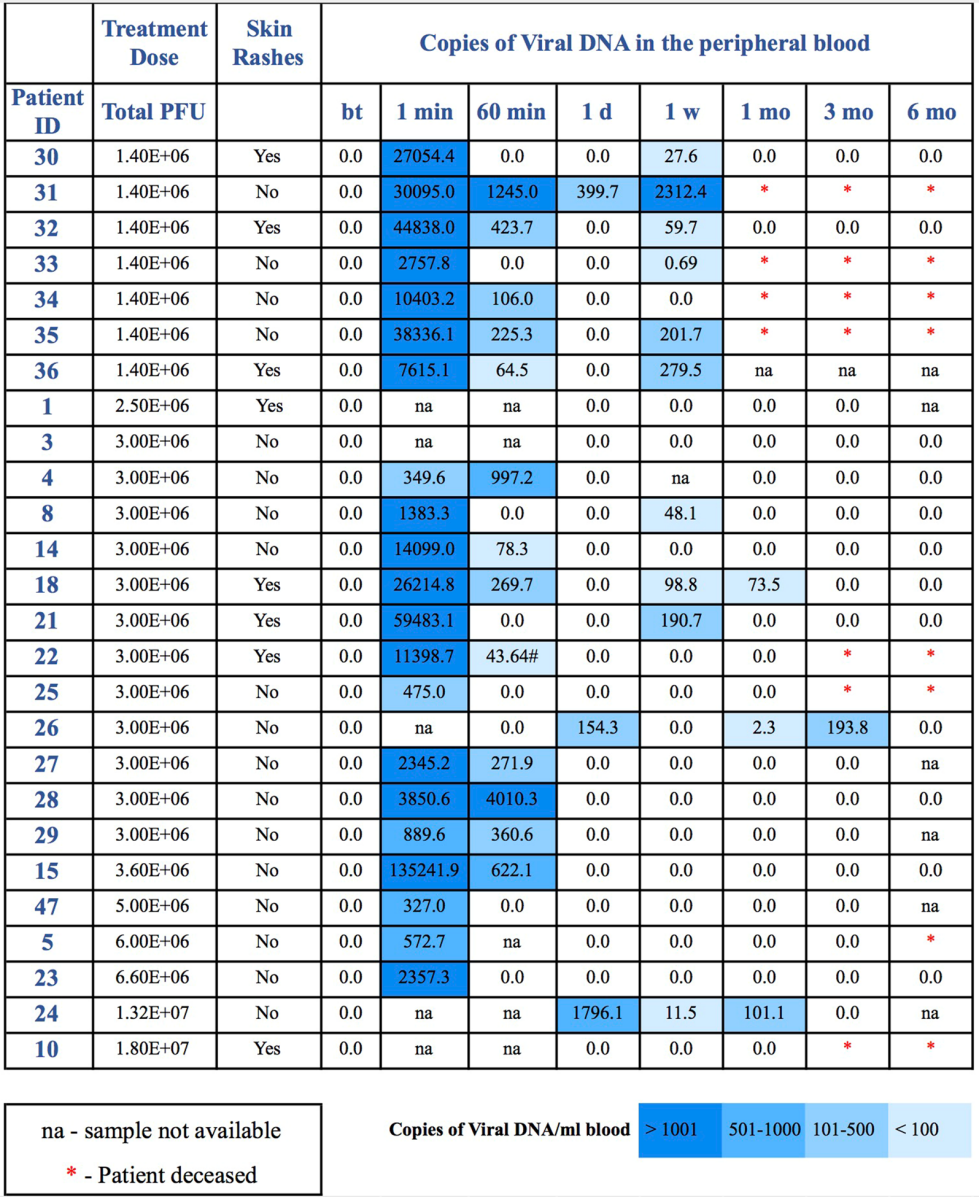
Viral DNA in patients’ peripheral blood. DNA was extracted using the Quick-gDNA™ Blood MidiPrep (Zymo Research, CA). The copy number amount of the ACAM2000 gene A56R was quantified by qPCR using PowerUp™ SYBR^®^ Green Master Mix (Thermofisher, CA). Viral DNA was analyzed by qPCR before treatment (bt), and 1 min (1 min), 60 min (60 min), 1 day (1d), 1 week (1 w), 1 month (1 mo), 3 months (3 mo) and 6 months (6 mo) after treatment

In 10 out of 24 patients, ACAM2000 DNA was present in peripheral blood 1 week post treatment (Fig. 2). In 8 out of those 10 patients, viral DNA was not detected on day 1 post treatment. In 1 of those 10 patients, viral DNA was lower on day 1 than on day 7 post treatment. These results indicate that viral DNA present 1 week after treatment might be originating from ACAM2000 active replication, probably at tumor sites. In 3 out of 21 patients, ACAM2000 DNA was present in peripheral blood 1 month post treatment. In one of those 3 patients, ACAM2000 DNA was present 3 months post treatment. No ACAM2000 DNA was detected 6 months post treatment in any patient.

### Efficacy assessment

**Fig. 3:**
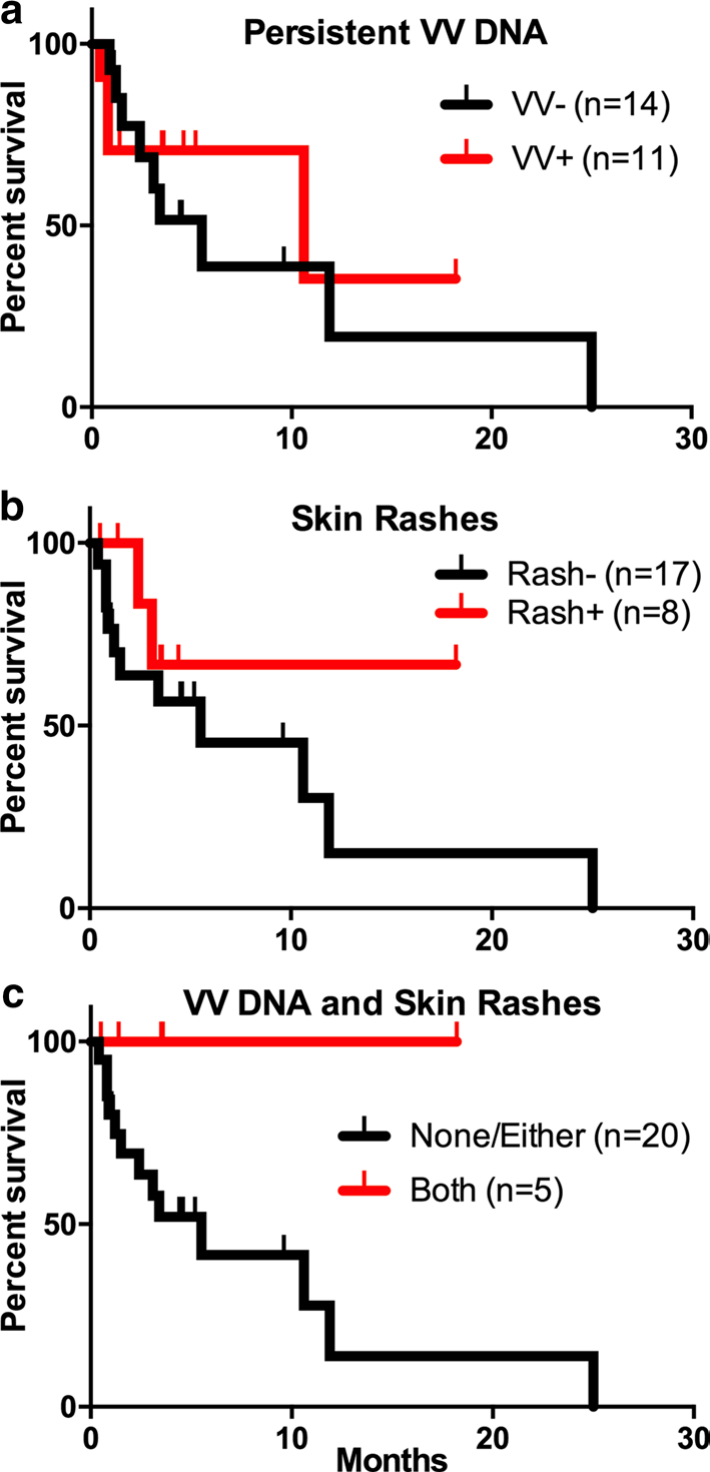
Kaplan-Meier plots showing the relative survival of a total of 25 patients (23 patients with solid tumors and 2 patients with AML). **a** Patient survival relative to the presence of persistent vaccinia virus DNA in the blood for 1 week to 3 months post treatment. Median survival: without persistent VV, 5 months, (n = 14) vs. with persistent VV, 10 months (n = 11). **b** Patient survival relative to reported presence of skin rashes. Median survival: without skin rashes, 5 months (n = 17) vs. with skin rashes, undefined (n = 8). **c** Patient survival relative to the presence of both skin rashes and persistent vaccinia virus DNA in the blood. Median survival: with either or none, 5 months, (n = 20) vs. with both, undefined (n = 5). Vertical tick marks indicate patients who dropped from the study or were censored from analysis after switching to alternative cancer treatments

An important observation in this study was that patients with evidence for oncolytic virus activity, including persistance of viral DNA in the blood from 1 week to 3 months following treatment, or having skin rashes, demonstrated trends towards longer survival (Fig. 3). The patients who presented with both persistent viral DNA in the blood and skin rashes showed strongest trend towards longer survival (Fig. 3c). For the 11 patients with persistent viral DNA in the blood, the median survival was 10 months, compared to only 5 months for the patients without persistent viral DNA in the blood. However, despite these results, the survival curves were not statistically significantly different from one another. This is due to the limited number of patients and/or differences in disease type/progression that preclude proper and conclusive evaluation of therapeutic benefits at this stage. A median survival period of 5 months was also calculated for the sub-cohort of 17 patients in whom no skin rashes were detected (Fig. 3a), although it should be emphasized again that no statistically significant differences were noted amongst the survival curves.

The main purpose of this Phase I clinical study was to establish the safety and tolerability of ACAM2000/SVF, as this approach is being tested for the first time in patients with advanced solid tumors and AML. However, we were able to assess some initial efficacy signals in several patients.

**Fig. 4:**
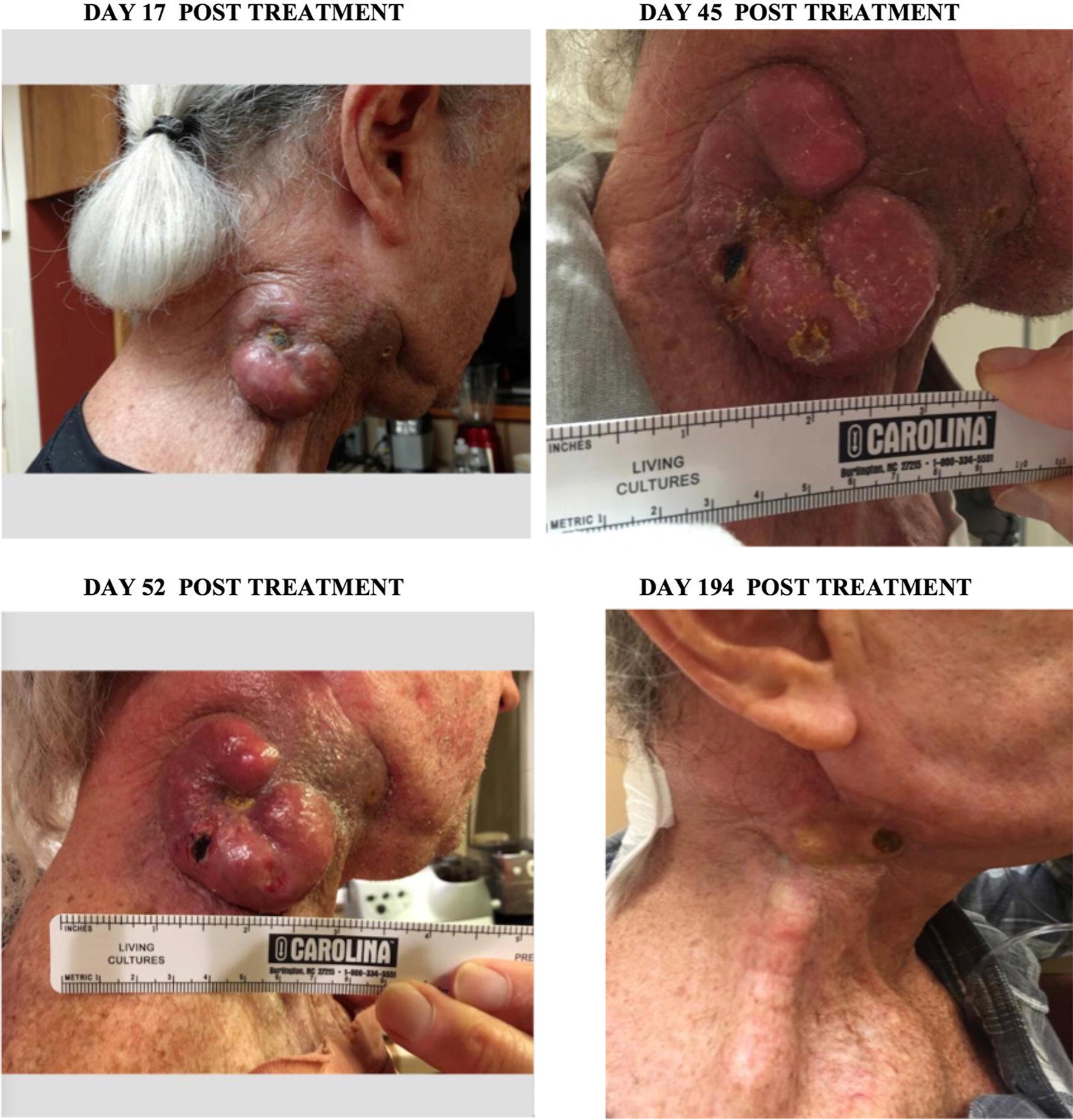
Patient #21: tumor regression of patient’s poorly differentiated squamous cell carcinoma (Stage: IV_B)

Patient #21 was 70 years old male diagnosed with metastatic poorly differentiated squamous cell carcinoma (Stage: IVB). Patient presented with a very large tumor near right ear heading toward collar bone and other smaller tumors on the opposite side. Patient previously received XRT to right lesion as well as Paclitaxel and 5FU. Treatment with ACAM2000/SVF was divided in 4 independent inoculations performed the same day: an IV infusion of ACAM2000/SVF and 3 IT injections into three regions of his right neck tumor. Three weeks post treatment the patient reported itchiness, warmth and slight enlargement of treated lesion as well as some oozing (Fig. 4). Biopsy of this lesion at 4 weeks post treatment showed poorly differentiated squamous cell carcinoma arranged in sheets and groups with comedo-type necrosis, inflammation and surrounding fibrovascular stroma. Six weeks post treatment patient began treatment with Opdivo (Nivolumab, anti-PD-1) q2w 3 mg/kg. Three months post ACAM2000/SVF treatment this patient received XRT (13 doses) accompanied with fatigue, nausea, and difficulty swallowing. Approximately 2 weeks after completion of XRT treatment the tumor began shrinking, leading to a substantial size reduction at 6 months post ACAM2000/SVF treatment, weight gain and significant improvement of overall condition (Fig. 4).

**Fig. 5:**
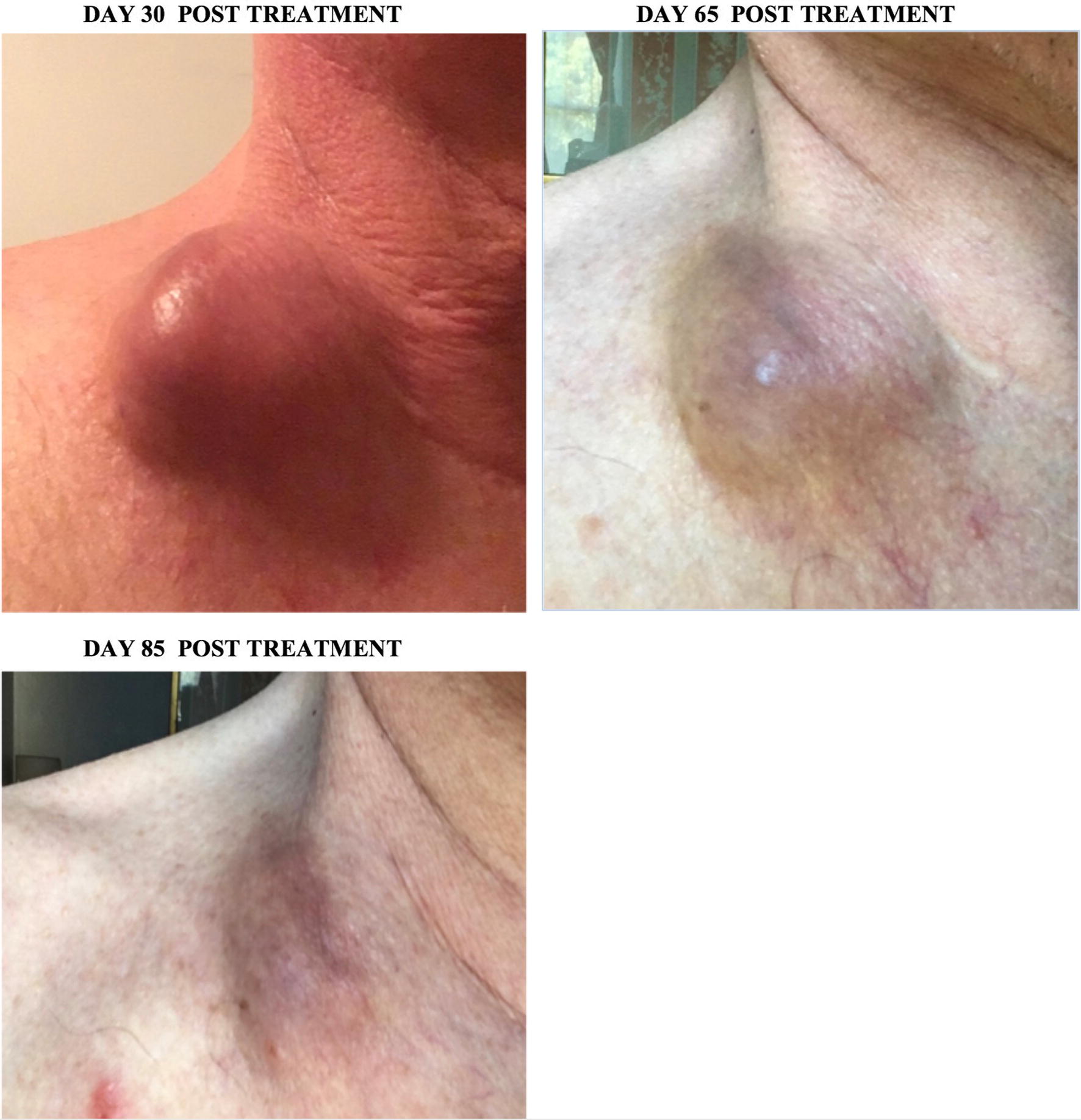
Patient #47: tumor regression of patient’s metastatic papillary thyroid carcinoma. Treatment effects in the treated right supraclavicular lymph node are shown

Patient #47 was 67 years old male diagnosed with metastatic papillary thyroid carcinoma with cervical lymphadenopathy. Left thyroidectomy and isthmectomy was done in 1985. Patient presented with a large clearly visible right supraclavicular lymph node, a smaller left cervical node and several smaller palpable cervical nodes. Treatment with ACAM2000/SVF was divided in 6 independent intra-tumoral inoculations performed the same day: 4 IT injections into four regions of the larger right supraclavicular lymph node and 2 IT injections into two regions of the smaller cervical lymph node. Ipilimumab (anti-CTLA-4) was injected 36 h post ACAM2000/SVF injections as a single IT injection of 25 mg Ipilimumab into the right node only. Approximately 4 weeks post treatment the patient reported enlarged, warmer and reddish right node and no inflammatory symptoms in the left node (Fig. 5). Two months post treatment the right tumor was much smaller, softer and much less inflamed, while no changes in consistence or appearance were noted in the left treated node. Three months post treatment the patient experienced almost complete eradication of the right tumor with a very small hard area notable only on palpation (Fig. 5).

## Discussion

This Phase I clinical study was designed to establish the safety and tolerability of ACAM2000/SVF and assess initial efficacy signals in relationship to ACAM2000/SVF treatment. The most important finding of this study was that ACAM2000/SVF can safely be administered in patients with advanced metastatic solid tumors or advanced AML. Two important aspects of this finding will have clear clinical implications in future trials: (i) this is the first clinical study confirming the safety of a TK-positive oncolytic vaccinia virus delivered by autologous cells, and (ii) safety administration of ACAM2000/SVF is confirmed in severely immunocompromised patients with advanced cancer. This trial also validates the safety of combining ACAM2000 and SVF as a delivery vehicle that protects the virus from complement inactivation in the blood. No significant treatment-associated toxicities were observed in any of the 26 patients who received IV, IP and IT injections of ACAM2000 loaded onto freshly isolated SVF, showing that ex vivo infection of the SVF can be done in a manner that is safe to the patients.

Our pharmacokinetics data indicate that the injected virus (viral DNA detected by qPCR) is rapidly cleared from blood circulation within an hour. In some patients the viral DNA reappeared 1 week to 1 month following treatment, and importantly often in the absence of any skin rashes which suggests intra-tumoral virus infection and amplification. Despite the potential for sustained intra-tumoral virus amplification, none of the treated patients developed uncontrolled viremia or manifested widely spread skin rashes or any other evidence for systemic toxicity, supporting our conclusion that intra-tumoral virus amplification does not pose a significant safety concern.

AEs associated with smallpox vaccination in general are fever, headache, myalgia, rigors, sweating, fatigue, asthenia, nausea, vomiting, general reddening of the skin, lymph node swelling, and immunological parameter changes (e.g., cytokines). In the past, various vaccinia virus strains (DryVax, Lister) have been applied clinically to cancer patients [13, 14, 27, 28]. Positive treatment results have been reported, along with some severe local reactions (rash, erythema) as well as systemic side effects (headache, malaise, fever, flu-like symptoms), but no life-threatening toxicities. These previously published results provided the rationale and scientific justification for us to conduct further clinical investigation of the oncolytic potential of ACAM2000, the current plaque-purified and naturally attenuated smallpox vaccine. The most concerning toxicities observed with the use of ACAM2000 as a smallpox vaccine are rare cardiac complications (arrhythmias, pericarditis, myocarditis, and dilated cardiomyopathy). No cardiac complications or systemic side effects were observed in any patient participating in the current trial. This finding contrasts with previous clinical trials with “naked” vaccinia viruses, where most patients experienced fever, chills and other flu-like symptoms for the first 6–24 h following treatment [13, 14].

We have demonstrated that ACAM2000 is oncolytic and efficiently infects and kills human cancer cell lines, while the extensive use of the same virus as a smallpox vaccine has conclusively demonstrated self-limiting amplification at the site of administration in the skin with virtually absent local or distant spread to other tissues and organs in the body [15]. These observations likely reflect the combination of efficient neutralization in circulation and the inability of the virus to infect and amplify in healthy cells, apart from the limited amplification seen in skin keratinocytes. This study confirmed the previous observations with various heterogeneous and more virulent smallpox vaccines previously licensed in the USA and Europe that the AEs associated with the application of smallpox vaccines to cancer patients are minor and rarely require medical attention [27, 28]. Overall, the observed treatment-related adverse events in the study were rare. These findings suggest that ACAM2000 is well tolerated and can be safely administered to patients with cancer.

Despite this positive safety data, the investigator administering ACAM2000 must be prepared for extremely rare but possible severe local and systemic reactions, historically associated with patients who had inherent or treatment-related immunodeficiency. In the unlikely scenario that such complications do occur, the adverse events and complications can be effectively controlled by the available antidote vaccinia immune globulin (VIG) [29]. Although cardiac toxicities were not reported by any patient in our safety study, specific efforts should be taken to closely monitor the cardiac condition of all patients undergoing ACAM2000 treatment in an effort to detect such symptomatic or asymptomatic virus-related complications and prevent possible cardiac events by timely administration of the antidote, if considered necessary by the monitoring physician.

The therapeutic potential of any oncolytic virus depends on the fine balance between the induction of antiviral immunity, leading to clearance of the oncolytic virus, and the development of antitumor immunity, leading to tumor cell eradication and eventually to potent and durable clinical responses. The current trial represents an attempt to fine tune this balance by protecting the oncolytic virus using autologous SVF cells to avoid premature clearance of the virus, allowing sufficient time for viruses to replicate and kill tumor cells and to initiate antitumor immunity. Interestingly, the analysis of this safety trial suggests that there is no positive correlation between augmented vaccinia virus activity and increased tumor burden, tumor-associated immunosuppression, and shorter survival. Overall, the trends towards improved survival associated with vaccinia virus activity in vivo including the persistence of viral DNA in the blood, visible skin rashes, and/or both (Fig. 3), are intriguing and may indeed have therapeutic significance.

The two case reports presented here emphasize the importance of combining checkpoint inhibitors and oncolytic viruses to achieve better clinical responses. The synergistic effects of this combination have been described previously by us [30] and other groups [31, 32]. Therefore, these current trial findings will be utilized in future clinical trial designs with this synergistic approach.

When evaluating the efficacy outcomes of the current study, it is important to note that the primary

objective of this study was to evaluate the safety of the combination of ACAM2000 and SVF in patients with advanced tumors. Therefore, we acknowledge that the interpretation of the efficacy outcomes is limited by the small size of the study population (n = 26). Only a randomized trial would be able to definitively demonstrate the efficacy of the ACAM2000/SVF combination.

## Conclusions

The aggregate safety, tolerability, and PK results indicated that ACAM2000/SVF was well tolerated in this study with 26 patients with advanced cancers (Stage III or IV). The MTD was not reached.

In summary, our study show that: (i) the combined application of SVF and ACAM2000 was very safe in all patients; (ii) the results of the plasma cytokine assays suggested mild inflammatory reaction starting approximately 1 week after treatment, not associated with any clinical symptoms; (iii) most patients experienced virus-related and inflammation-related symptoms at the tumor sites approximately 2 weeks after treatment; (iv) the flow cytometry assays show induction of immune response with memory T cells approximately 1 month after treatment; (v) there is a trend towards improved survival associated with vaccinia virus activity in vivo including the persistence of viral DNA in the blood, visible skin rashes, and/or both; (vi) some patients experienced significant tumor size reduction, especially when the ACAM2000/SVF treatment was combined with checkpoint inhibition.

These early promising results must be re-evaluated within a larger and more homogeneous cohort of patients to confirm the feasibility and effectiveness of this novel treatment approach.

## Data Availability

The datasets used and/or analyzed during the current study are available from the corresponding author on reasonable request.

## Abbreviations

AML: acute myeloid leukemia
AE: adverse event
AJCC: American Joint Committee on Cancer
CTCAE: Common Terminology Criteria for Adverse Events
CTLA-4: cytotoxic T-lymphocyte antigen 4
MOI: multiplicity of infection
PD-1: programmed death 1
PFU: plaque-forming units
qPCR: quantitative PCR
RECIST: response evaluation criteria for solid tumors
SVF: stromal vascular fraction
TK: thymidine kinase
VV: vaccinia virus
